# School Health Services’ Use of Information and Communication Technologies in Interorganizational Collaboration Regarding Students With Mental Illness: A Scoping Review

**DOI:** 10.1177/10598405241245029

**Published:** 2024-04-09

**Authors:** Angelika Johansson Cristvall, Margaretha Larsson, Johanna Tell, Lisa Skär

**Affiliations:** 1 Institution of Health, Blekinge Institute of Technology BTH, Karlskrona, Sweden; 2 Institution of Health and Learning, University of Skövde HIS, Skövde, Sweden; 3 Dean Faculty of Health Science, Kristianstad University HKR, Kristianstad, Sweden

**Keywords:** adolescents, cooperation, digital technology, health information technology, mental health, school health, school nurse, system collaboration

## Abstract

School health services (SHSs) and school nurses play a crucial role in identifying and supporting students with mental illness. The integration of information and communication technology (ICT) can facilitate interorganizational collaboration in this context. Due to the limited research in this area, a scoping review was conducted to explore SHSs’ use of ICT in interorganization collaboration regarding students with mental illness. Six articles were reviewed, revealing three key themes: “types of ICT employed by SHSs in interorganizational collaboration,” “constellation of SHSs in interorganizational collaboration,” and “opportunities and challenges for SHSs using ICT in interorganizational collaboration.” Notably, two of the six articles highlighted the absence of school nurses in interorganizational collaboration. Even though ICT plays a crucial role in interorganizational collaboration, no comprehensive solution was found. This scoping review confirms that there are challenges with operability and regulations that govern the exchange of private information between organizations.

## Introduction

School health services (SHSs) and school nurses play a crucial role in identifying and supporting students with mental illness. School health personnel collaborate with others both in the school systems and with others in the community to assist students with mental and behavioral health issues. Often, these collaborations require complex information exchange and are subject to communication gaps and disruption. Information and communication technology (ICT) can facilitate interorganizational collaboration in this context. The purpose of this article was to explore SHSs’ use of ICT in interorganization collaboration regarding students with mental illness.

Adolescence is a period marked by identity exploration, encompassing physical, social, intellectual, and emotional changes, making it a challenging developmental phase (Livesey & Ronstain, 2017). Adolescence is a transitional stage from childhood to adulthood, spanning the age range of 10–19 years (World Health Organization [WHO], 2021). Mental illness often emerges before the age of 18 in half of adolescents facing such challenges (Kelly et al., 2011). For adolescents, mental health problems and mental illnesses can significantly affect daily functioning (Granlund et al., 2021), particularly in settings like school. Research shows that early interventions for mental illness can alleviate personal suffering and reduce societal costs by decreasing the need for subsequent interventions (Kaushik et al., 2016). The mission of SHSs is to support the health and learning of students, delivered by health professionals either on school premises or through external health services (WHO, 2021). In Sweden, SHSs are free of charge and voluntary for users and include professionals such as school physicians and nurses, psychologists, social workers, and special education teachers, according to the Swedish Education Act (SFS 2010:800).

School nurses play a central role in identifying and supporting students with mental illnesses that might hinder their educational progress (Bohnenkamp et al., 2015; Dina & Pajalic, 2014; Lyon et al., 2019). According to the Swedish Education Act (SFS 2010:800), all students are offered a special health conversation with a school nurse three times up to grade 9, covering elementary and middle school, and one appointment during high school. These mandated health visits include conversations covering various lifestyle factors such as diet, exercise, sleep, substance use, puberty, sexuality, and psychosocial aspects. The aim is to promote health with a focus on the student's needs, identify and prevent issues, and build a trusting relationship between the school nurse and student for future support (Socialstyrelsen, 2016). In Sweden, school nurses are bound by a duty of confidentiality (SFS 2009:400). Research indicates that these health dialogues, conducted by school nurses, significantly contribute to enhancing students’ health (Larsson et al., 2013) and aid in achieving their educational goals (Kostenius, 2023). School nurses have the skills to serve as leaders and coordinators for SHSs. In line with the school nurse competencies they assess, plan, implement, and evaluate measures aimed at promoting physical, mental, and social health (National Association of School Nurses, 2016). They play a central role by collaborating with other professionals within the school health as school physicians, teaching staffs, community healthcare providers and can be seen as the “spider in the web” in coordinating collaboration between different actors within and outside the organization (Gädda et al., 2023). They hold a unique position conducive to facilitating communication between SHSs and other healthcare providers (Leroy et al., 2016). Even though school nurses possess skills that prepare them for collaboration within their organization, among different professions, and in interorganizational collaboration, there is room for improvement in the communication between professionals (Bohnenkamp et al., 2015). Interorganizational collaboration, as explained by Lee et al. (2013), is an interpersonal process where members of different disciplines contribute to a shared product or common goal. Schools could serve as environments fostering interorganizational collaboration, particularly with other services for children and adolescents with mental illness as severe emotional disorders. Collaborative care, characterized by a multi-professional approach to patient care, involves a structured management plan, scheduled patient follow-up, and enhanced interprofessional communication (Gunn et al., 2006). The use of ICT in SHSs can be described with various terms (Zonneveld et al., 2019). The World Health Organization (WHO) uses the term eHealth to denote the use of ICT to support population health and in healthcare areas (WHO, 2005). The term ICT was chosen in this study to encompass all technologies utilized SHS in interorganizational collaboration. Furthermore, implementing and disseminating ICT in SHSs facilitates collaboration among professionals working in distant locations. This collaboration between SHSs and mental health services could enhance access to specialized care for vulnerable adolescents (Pradhan et al., 2019). The WHO (2023) underscores the safety, cost-effectiveness, and crucial role of ICT in achieving overall health priorities, including efforts in SHSs. ICT enables greater accessibility to healthcare (Myers & Cain, 2008) and saves time for both healthcare providers and patients (Haleem et al., 2021). Obstacles to using ICT in interorganizational collaboration include challenges such as interoperability, which refers to the ability of different systems, technologies, or components to work together (Auschra, 2018). While digitization has made significant progress in Sweden in other industries, healthcare lags behind (The Swedish Agency for Digital Government [DIGG], n. d.). The Swedish government's investigation (SOU 2021:78) reveals that deficiencies in information sharing affects interorganizational collaboration. It is vital to seamlessly share information across systems, among professionals within schools, and with communities (Bohnenkamp et al., 2015). However, sharing students’ information is hindered by technical challenges (Allen et al., 2020). Legislation related to data protection can be viewed as an obstacle to sharing personal information. For example, the General Data Protection Regulation (GDPR) can make sharing personal information less effective and more complex (Feinstein et al., 2023). Given the advantages of schools’ interorganizational collaboration with community-based professionals, it is essential to address potential ethical issues and communication problems that might hinder such collaboration (Zoromski et al., 2015).

## Methods

To explore SHSs’ use of ICT in interorganization collaboration regarding students with mental illness a scoping review was conducted as it proves to be a valuable method for synthesizing evidence and assessing the breadth of literature on a specific topic (Arksey and O’Malley, 2005). The preparation of this article adhered to the Preferred Reporting Items for Systematic Reviews and Meta-Analyses (PRISMA), specifically tailored for Scoping Reviews, known as PRISMA-ScR (PRISMA, 2018). The analytical method outlined by Arksey and O’Malley (2005) was followed. To formulate the research questions, the assistance of an experienced librarian was sought. This collaboration aimed to select appropriate search terms for databases and construct search strings in accordance with the guidelines provided by Arksey and O’Malley (2005).

### Eligibility Criteria

The articles included in the review focused on SHSs or similar health services affiliated with schools, exploring interorganizational collaborations and interactions with professionals outside of schools regarding students with mental illness from first to twelfth grade. The selected articles specifically investigated the utilization of ICT in this context and were presented in the English language. The design of the included articles encompassed qualitative and mixed methods.

### Information Sources

**Figure 1.**
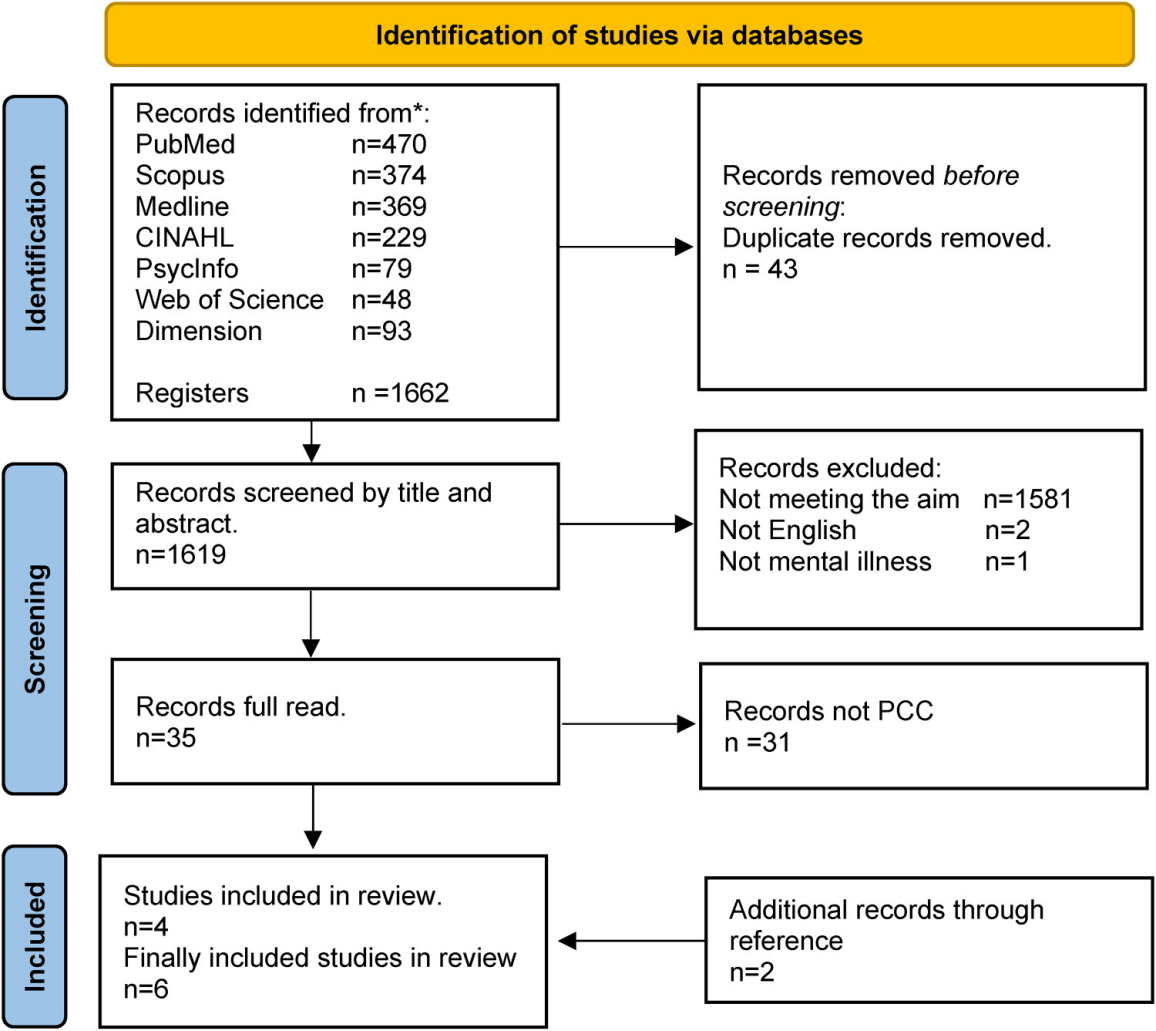
PRISMA flowchart of search and inclusion.

Relevant information for the study was systematically searched across various databases suitable for the subject area (Polit & Beck, 2017). The selected databases for this review included Medline, Scopus, PubMed, CINAHL, PsycINFO, Web of Science, and Dimension. The platform for Medline and CINAHL was EBSCO. To refine the search, a set of keywords and synonyms were used, such as “interorganizational,” “collaboration,” “school health services,” “school nurse,” “students,” “adolescence,” “mental illness,” and “information and communication technology.” Boolean operators (OR and AND) were employed, and truncation (*) was used to enhance search sensitivity. The searches were conducted in March 2023, yielding a total of 1662 articles. The articles were organized and managed using Zotero, a reference management program, ensuring accuracy and quality of source documentation (Karolinska Institutet, 2023). An experienced librarian assisted in compiling seven search strings, each tailored to the specific search rules of the respective database. One of the seven search strings can be found in Table 1. After removing duplicates, the titles and abstracts of the remaining articles were reviewed, and those not aligning with the study's aim were excluded. Subsequently, all articles underwent a thorough examination, applying the population, concept, and context (PCC) framework to identify and exclude articles that did not contribute to the research objectives (University of South Australia, 2023). Additionally, two relevant articles were discovered through references in the included articles, see Figure 1. Finally, six articles were included in the scoping review.

**Table 1.** Search String PubMed 31 of March 2023


(organi* OR interorgani* OR organization OR organizations OR organisation OR organisations OR interorganizational OR interorganisational OR “inter organizational” OR “inter organisational” OR “inter-organizational” OR “inter-organisational” OR interinst* OR interinstitutional OR interprof* OR interprofessional OR “inter institutional” OR “inter professional” OR “inter-institutional” OR “inter-professional” OR intersector* OR intersectoral OR “inter sectoral” OR “inter-sectoral” OR “child and adolescent psychiatry” OR CAP OR “social services” OR collab* OR collaborate OR collaborating OR collaborates OR collaboration OR cooperat* OR cooperate OR cooperating OR cooperation OR “co-operate” OR “co-operates” OR “co-operating” OR “co-operation”) AND (“school health” OR “school nursing” OR “school nurse” OR “school nurses” OR “school mental”) AND (mental* OR mental OR mentally OR psych* OR psychological OR psychology OR psychiatric OR psychiatry) AND (ICT OR “information technology” OR “communication technology” OR “communications technology” OR digital* OR digital OR digitalization OR digitalisation OR digiti* OR digitization OR digitisation OR comput* OR computer OR computers OR online* OR online OR internet* OR internet OR web OR WWW OR smartphone* OR smartphone OR smartphones OR “smart phone” OR “smart phones” OR iphone* OR iphone OR iphones OR mobile* OR mobile OR mobiles OR cellphone OR cellphones OR cellphone* OR “cell phone” OR “cell phones” OR “cellular phone” OR “cellular phones” OR tablet* OR tablet OR tablets OR ipad* OR ipad OR ipads OR software* OR software OR softwares OR app OR apps OR application OR applications OR eHealth OR “e-health” OR “e health” OR “electronic health” OR informatics)

### Data Charting

Data extraction was carried out in both tabular and charting formats, with continuous updates made in an iterative process. The first author, A, included and charted the data and discussed the results with the second author, B. Both authors re-read the articles separately to appraise each for inclusion in the scoping review. Subsequently, both authors independently excluded additional articles that did not sufficiently describe the use of ICT. The extracted data were charted and organized by title, origin country, year of publication, method, analytical approach, aim, and outcome based on the research question, along with information on ethics, limitations, funding, and the journal, see Table 2. The articles were further grouped and summarized by country, population, year, method, ethics, limitation, funding, and journal. Synthesized data addressing how ICT was employed in SHS intercollaboration regarding students with mental illness were charted, compiled in an Excel document, and subsequently thematically analyzed. All data were reviewed to identify similarities and differences linked to the research question, leading to the emergence of three themes.

**Table 2.** Charting of Articles Included in the Qualitate Cleematic Analyze.

Author(s), publication, location	Aim	Design, method, and carer group	Important findings
Bates, S. M., Mellin, E., Mellin, L. M., Anderson-Butcher, D., Vogeler, M. & Sterling, K. 2019 USA/Intermountain West Journal; *Children & Schools*	To explore interprofessional team collaboration practices and processes and to examine whether student-level outcomes are associated with interprofessional team collaboration.	A mixed method with semi-structured interviews was analyzed thematically, and a quantitative part, where paired sample t-tests examined data, involved a total of 340 students from 4 elementary schools	A collaborative interprofessional care team. A care model, informed processes and structure and an inclusive linkage protocol was established to support mental health integration. Participants noted that technology played a key role.
Grady, B. J., Lever, N., Cunningham, D. & Stephan, S. 2011 USA/Maryland Journal; *Child and Adolescent Psychiatric Clinics of North America*	To prevent the nonpublic placement of children and adolescents with emotional disorders by providing more intensive support and school mental health services.	A mixed method involving focus groups and questionnaires was used to study children and adolescents with emotional disorders. The total number and age were not mentioned.	Coordinated mental health services and support in the school setting were video consultations, perceived as innovative and advanced technology.
Jahans-Baynton, K. & Grealish, A. 2021 England/London Journal: *Journal of Child and Adolescent Psychiatric Nursing*	To explore professionals’ views and experiences of safeguarding communication and identify areas for improvements to practice.	A descriptive qualitative design with semi-structured in-depth face-to-face interviews, and data were analyzed thematically. The participant group included children and adolescents. The total number and age were not mentioned.	Difficulties when using technology to communicate. It was perceived as time-consuming and frustrating. A shared system can improve communication across different agencies and boroughs.
Lyon, A. R., Knaster Wasse, J., Ludwig, K., Zachry, M., Eric J. Bruns, E. J., Unutzer, J. & McCauley, E. 2016(a) USA/Pacific Northwest Journal; *Administration and Policy in Mental Health and Mental Health Services Research*	To present data from the application of CTAP Phase 1, to the redesign of a Measurement Feedback System (MFS) for use by school-based mental health (SBMH) providers.	A qualitative design where data were collected via focus groups. The current project was part of a larger initiative involving middle schools and high schools in a large urban school district. The total number was not mentioned.	Limitations existed when clinics were not agents of the district and did not have access to the existing district education data system.
Lyon, A. R., Whitaker, K., French, W. P., Richardson, L. P., Knaster Wasse, J., & McCauley,E. 2016(b) USA/Washington Journal; *Advances in School Mental Health Promotion*	(a) Explore the potential usefulness of a Collaborative Care (CC) model to facilitate improved access, service integration, and quality in School Base Mental Health (SBMH); (b) Articulate a CC informed model to enhance School Base Mental Health (SBMH) services; and (c) Outline a research agenda to further advance CC in schools.	A mixed methods research design. The method of data collection and the total number and age were not mentioned.	A well-defined collaborative care model, specifically designed for schools, is an essential step in developing or adapting technologies to support it. Individuals with existing expertise working with multiple systems and integrating physical health, mental health, and educational health, individuals such as school nurses are suited for the role of a care manager.
Stephan, S., Lever, N., Bernstein, L., Edwards, S. & Pruitt, D. 2016 USA/Maryland Journal; *Journal of Child and Adolescent psychopharmacology*	To describe the potential and limits of school telemental health (TMH) in supporting a full continuum from mental health promotion to intervention, particularly for students less likely to access community care.	A mixed method, with interviews and focus groups, and a review of school TMH literature and model programs, was employed. The participant group consisted of schoolchildren. The total number and age were not mentioned.	TMH services include advantages such as increased efficiency, higher volume capacity, and increased access to care. A hybrid model of care involving both in-person and telemental health care was proposed as a solution

## Results

### Overview

In this scoping review, six articles were identified, all addressing the research question of SHSs’ use of ICT in interorganization collaboration regarding students with mental illness. The articles were published between 2011 and 2022, with five originating from the United States (Bates et al., 2019; Grady et al., 2011; Lyon, Knaster Wasse, et al., 2016, Lyon, Whitaker, et al., 2016; Stephan et al., 2016) and one study conducted in England (Jahans-Baynton & Grealish, 2021). The design was a mixed method in four articles (Bates et al., 2019; Grady et al., 2011; Lyon, Whitaker, et al., 2016; Stephan et al., 2016) and qualitative in two articles (Jahans-Baynton & Grealish, 2021; Lyon, Knaster Wasse, et al., 2016). Only one study provided the total number of students involved (Bates et al., 2019), and ethical considerations were discussed in one article (Bates et al., 2019). Funding details were discussed in two articles (Lyon, Whitaker, et al., 2016; Stephan et al., 2016), and limitations were addressed in two (Bates et al., 2019; Lyon, Knaster Wasse, et al., 2016). All articles were published in different journals. It is notable that none of the articles directly addressed the review question regarding the exploration of SHSs’ use of ICT in interorganization collaboration regarding students with mental illness. The summarized results are presented in three themes: types of ICT employed by SHSs in interorganizational collaboration, constellation of SHSs in interorganizational collaboration, and opportunities and challenges for SHSs using ICT in interorganizational collaboration.

### Types of ICT Employed by SHSs in Interorganizational Collaboration

The compiled types of ICT used by SHSs in interorganizational collaboration to support students with mental illnesses found in the results cover a wide range of technological solutions in a fragmented way. No overall solution adapted for SHSs in interorganizational collaboration was found. Types of ICT include telehealth and audio and video consultations with a psychiatrist to consult professionals in SHSs and students (Grady et al., 2011; Stephan et al., 2016). Bates et al. (2019) explored interprofessional team collaboration practices through a school's identification, referral, and intervention system to assess students’ needs, collaborate with parents or caregivers, and refer students to the care team for further assistance. Telehealth is one form of telepsychiatry that focuses on consultations with experienced psychiatrists for professionals in SHSs through audio and video consultations (Grady et al., 2011). Another form is telemental health (TMH) services, which aim to provide mental health support to adolescents in schools and make psychiatric care in schools more feasible by facilitating collaboration among psychiatrists, educators, and other health and mental health professionals (Stephan et al., 2016). Bates et al. (2019), Grady et al. (2011), and Stephan et al. (2016) were all based on a program from the University of Maryland Department of Psychiatry. Another study explored safeguarding communication, including the Multi-Agency Safeguarding Hub (MASH), a team that collects and shares identifiable patient information between professionals. The article did not describe the technology and information management systems used, except for secure email (Jahans-Baynton & Grealish, 2021). Lyon, Whitaker, et al. (2016) capitalize on the synergy between collaborative care and multi-tiered systems of support (MTSS). The project was titled Accessible, Collaborative Care for Effective School-based Services (ACCESS), which aims to retain the essential components of collaborative care and adapt them to an educational context. One key issue this model addresses is how integrated data systems support collaborative care delivery within the education sector. An essential aspect is tracking educational and health-related data, which may necessitate developing new technologies or adapting existing ones. Due to challenges in implementing ICT in different healthcare settings across contexts, Lyon, Knaster Wasse, et al. (2016) propose the Contextualized Technology Adaptation Process (CTAP) to guide the design of technologies to ensure high compatibility with a destination setting. For this project, an existing measurement feedback system (MFS), the Mental Health Integrated Tracking System (MHITS), was selected and integrated into a collaborative process that involved collaboration between school-based providers and externals. MHITS is a measurement feedback system designed to manage quantitative data and deliver automated presentations of information, facilitating swift and clinically valuable feedback to mental health providers (Lyon, Knaster Wasse, et al., 2016). Other technologies mentioned alongside the technology described above include Google Docs (Bates et al., 2019), secure email (Jahans-Baynton & Grealish, 2021), and unspecified forms of texting and social media for communications related to service delivery (Lyon, Knaster Wasse, et al., 2016).

### Constellation of SHSs in Interorganizational Collaboration

All collaborations in the concluded articles involved professionals from various teams within SHSs, as well as external organizations such as public-health services (Jahans-Baynton & Grealish, 2021; Lyon, Knaster Wasse, et al., 2016) and community providers (Lyon, Knaster Wasse, et al., 2016; Stephan et al., 2016), with different professionals and organizations within those organizations as community mental health professionals (Bates et al., 2019), psychiatrists (Grady et al., 2011; Lyon, Whitaker, et al., 2016; Stephan et al., 2016), and primary care (Jahans-Baynton & Grealish, 2021; Lyon, Whitaker, et al., 2016). School nurses were mentioned as an integral part of the SHSs by Grady et al. (2011), Jahans-Baynton and Grealish (2021), Lyon, Whitaker, et al. (2016), and Stephan et al. (2016). Additionally, counselors were raised as appropriate professionals within the school health team in the articles by Grady et al. (2011), Lyon, Knaster Wasse, et al. (2016), Lyon, Whitaker, et al. (2016), and Stephan et al. (2016). Other mentioned professions in the school setting included, among others, principals (Bates et al., 2019; Lyon, Knaster Wasse, et al., 2016, Lyon, Whitaker, et al., 2016; Stephan et al., 2016), school psychologists (Bates et al., 2019; Grady et al., 2011; Lyon, Whitaker, et al., 2016; Stephan et al., 2016), and teachers (Grady et al., 2011; Jahans-Baynton & Grealish, 2021; Lyon, Knaster Wasse, et al., 2016; Stephan et al., 2016). In two articles (Grady et al., 2011; Stephan et al., 2016), the collaboration comprised of SHSs receiving support from child and adolescent psychiatrists. Bates et al. (2019) described having a lead case manager in their care team to oversee goal implementation. Lyon, Whitaker, et al. (2016) highlighted the importance of having a care manager in a care team, coordinating communication between service providers and recipients to arrange the services provided by various medical and mental health professionals, offering some lower-intensity mental health interventions, and monitoring outcomes. Individuals suitable for this role include school nurses, school social workers, and school psychologists, implying a potential need for them to acquire additional skills.

### Opportunities and Challenges for SHSs Using ICT in Interorganization Collaboration

There appeared to be both opportunities and challenges in SHSs’ use of ICT in interorganizational collaboration regarding students with mental illness. On the one hand, the results showed that technology is essential and plays a crucial role in processing and structuring interprofessional collaboration (Bates et al., 2019) and that ICT is a cornerstone due to the ability to track education and health-related data (Lyon, Whitaker, et al., 2016). Grady et al. (2011) stated that the ability to include video consultations enhanced schools by presenting an innovative program supported by state-of-the-art technology. On the other hand, concerns raised regarding ICT were about not having the capability to run modern software, changes or updates to technology products that decreased their ability to maintain mastery, and costs associated with purchasing new technology (Lyon, Knaster Wasse, et al., 2016). MASH emerged to support the sharing of personal information, but it was experienced as time-consuming and with a risk of sharing outdated information (Jahans-Baynton & Grealish, 2021). All participants in the study by Jahans-Baynton and Grealish (2021) encountered difficulties when using technology to communicate. As a result, challenges emerged from more superficial things, such as making telephone calls, which could be time-consuming (Jahans-Baynton & Grealish, 2021), to more complex challenges, such as sharing student information. It could be frustrating with different computer systems and security concerns sharing patient identifiable information (Jahans-Baynton & Grealish, 2021; Lyon, Knaster Wasse, et al., 2016). Face-to-face meetings were valued more highly than communication technologies in the study by Jahans-Baynton and Grealish (2021), and a hybrid model of care involving both in-person and telemental healthcare was proposed as a solution by Stephan et al. (2016). Ensuring effective communication for everyone is essential. Despite this, some healthcare information was not entirely shared among professionals due to privacy safeguards (Lyon, Whitaker, et al., 2016). Policies and processes could safeguard and improve safety in interorganizational collaboration, and a shared system could enhance communication across different organizations (Jahans-Baynton & Grealish, 2021).

## Discussion

Various ICTs were identified for use by SHSs but were implemented in a fragmented manner in interorganization collaboration regarding students with mental illness. The lack of a comprehensive technical solution for SHSs hindered effective collaboration across organizations. Technical challenges emerged, ranging from minor challenges such as time-consuming telephone calls, to more complex challenges associated with sharing students’ information. Previous research has highlighted the latter problem of sharing personal information (Allen et al., 2020; Auschra, 2018; Bohnenkamp et al., 2015; Feinstein et al., 2023; SOU, 2021, p. 78; Zoromski et al., 2015). It was previously recognized as crucial to seamlessly share personal information across systems (Bohnenkamp et al., 2015), and deficiencies in information sharing were acknowledged to affect interorganizational collaboration (SOU 2021:78). The findings confirm the ongoing challenges in sharing students’ information due to technical issues (Allen et al., 2020). No specific technical or legal solution addressing the interoperability problem was identified. The only organized and secure method for sharing students’ information between organizations that emerged was MASH, which acts as a third party and could be perceived as time-consuming and occasionally unreliable, with information becoming outdated. This area of how ICT could help SHSs facilitate better interorganizational collaboration, including sharing personal information regarding students with mental illness, could be valuable to explore further. The results showed that the school nurse was not part of SHSs and interorganizational collaboration in two of the six included articles. This is noteworthy, considering that earlier research highlights the importance of the school nurse's role in SHSs (Bohnenkamp et al., 2015; Dina & Pajalic, 2014; Kostenius, 2023; Larsson et al., 2013; Lyon et al., 2019). Research has shown that the health dialogues conducted by school nurses with adolescents play a crucial role in improving students’ health (Larsson et al., 2013) and contribute to reaching their educational goals (Kostenius, 2023). Earlier research also reveals that students also feel comfortable seeking assistance from school nurses when they are confident that their visits will remain confidential (Larsson et al., 2013), emphasizing the trusted and available nature of the school nurse as an essential element in student support systems (Kaskoun & McCabe, 2021).

School nurses can tactfully provide adolescents with tailored knowledge and health guidance, empowering individual students to actively participate in their health processes (Larsson et al., 2013). Despite this, Granrud et al. (2019) also highlight that school nurses are not always included in SHSs and their interorganizational collaboration regarding students with mental illness.

This study is the first to explore SHSs’ use of ICT in interorganization collaboration regarding students with mental illness, although technology is rapidly advancing across different sectors. Locating research that directly addresses the current objective has proven challenging, prompting the suitability of a scoping review. However, given that ICT is a broad field and healthcare services can be described using various terms (Zonneveld et al., 2019), it may be difficult to delineate the entire scope of the area.

ICT is a broad field of technology that is rapidly advancing across different sectors. It would be intriguing to explore school nurses’ perceptions of collaboration forms via ICT in interorganizational collaboration and how ICT could be designed to support interorganizational collaboration.

### Limitations

SHSs’ use of ICT in interorganization collaboration regarding students with mental illness is an area that is not well researched, so a scoping review is appropriate to scope previous research and identify (Arksey and O’Malley, 2005). Even during the scoping review, difficulties were encountered in finding articles explicitly addressing this area. Due to the difficulty of encompassing all technical terms related to ICT, there is a risk that the entire scope has not been thoroughly explored, potentially resulting in missed articles. The searches were conducted in March 2023, implying that additional articles could be discovered if the scoping review were repeated. A strength of this study is that keywords and the choice of databases were carefully considered with the help of an experienced librarian. The method is well described regarding verifiability, and search strings and databases are well documented, so the process can easily be redone. One author, A, performed the screening processes, and there is a risk that relevant articles were missed and that more relevant articles would have been found if there were several researchers in the screening process. It is a strength that author B was involved in the final step of selecting relevant articles, and both author B and author C participated in compiling the results. The inclusion of articles from different countries is beneficial, but a limitation is noted with five articles conducted in the United States, one in Europe, and a lack of representation from other countries. Furthermore, three (two describing research projects and one a program evaluation) of the six articles were based on a program from the University of Maryland Department of Psychiatry, potentially introducing bias and impacting the diversity and generalizability of the results. In just one of the four articles that employed mixed methods, they explicitly stated how they measured the quantitative part, paired sample t-tests examined data. This highlights another weakness identified within the literature.

## Conclusions

As a result, various types of ICT used by SHSs in interorganization collaboration regarding students with mental illness emerged as fragmented, with no comprehensive technical solution for the SHS setting. This highlights the importance of new technology in facilitating and organizing interprofessional collaboration. The results confirmed the presence of interoperability challenges stemming from different technological systems, as well as laws and regulations governing the exchange of private information between organizations. Furthermore, the study emphasizes the inclusion of school nurses in SHSs and interorganizational collaboration for students with mental illness, given their appropriate knowledge and skills.

### Implications for School Nurses

ICT has the dual capacity to both constrain and enhance interorganizational collaboration. It becomes crucial for school nurses to demonstrate curiosity and interest, influencing future technical solutions. Since school nurses serve as users of various technical solutions, their involvement in the development of these technologies is essential. Their contribution can offer valuable insights, particularly in aspects such as user-friendliness, even without extensive technical expertise. There is potential for investigating the development of a comprehensive ICT solution for SHSs and interorganizational collaboration regarding students with mental illness. This exploration could involve a school nurse serving as a coordinator and care manager, contributing to the creation of a platform aimed at improving communication and information exchange.

## Supplemental Material

sj-docx-1-jsn-10.1177_10598405241245029 - Supplemental material for School Health Services’ Use of Information and Communication Technologies in Interorganizational Collaboration Regarding Students With Mental Illness: A Scoping Review Supplemental material, sj-docx-1-jsn-10.1177_10598405241245029 for School Health Services’ Use of Information and Communication Technologies in Interorganizational Collaboration Regarding Students With Mental Illness: A Scoping Review by Angelika Johansson Cristvall, Margaretha Larsson, Johanna Tell, and Lisa Skär in The Journal of School Nursing
