# Supplementary material for: School Health Services’ Use of Information and Communication Technologies in Interorganizational Collaboration Regarding Students With Mental Illness: A Scoping Review
Source: J Sch Nurs. 2024 Apr 9;41(1):104–13. doi: 10.1177/10598405241245029 (PMC11755972; doi:10.1177/10598405241245029)
Supplement: sj-docx-1-jsn-10.1177_10598405241245029 - Supplemental material for School Health Services’ Use of Information and Communication Technologies in Interorganizational Collaboration Regarding Students With Mental Illness: A Scoping Review [file sj-docx-1-jsn-10.1177_10598405241245029.docx]

| Manuscript ID JSN-24-01-031 | |
| --- | --- |
| Table 1. Supplementary table for search strings | |
| **PubMed**  2023-03-21 |  |
| Inter-organizational collaboration | (organi* OR interorgani* OR organization OR organizations OR organisation OR organisations OR interorganizational OR interorganisational OR "inter organizational" OR "inter organisational" OR "inter-organizational" OR "inter-organisational" OR interinst* OR interinstitutional OR interprof* OR interprofessional OR "inter institutional" OR "inter professional" OR "inter-institutional" OR "inter-professional" OR intersector* OR intersectoral OR "inter sectoral" OR "inter-sectoral" OR "child and adolescent psychiatry" OR CAP OR "social services" OR collab* OR collaborate OR collaborating OR collaborates OR collaboration OR cooperat* OR cooperate OR cooperating OR cooperation OR "co-operate" OR "co-operates" OR "co-operating" OR "co-operation") |
|  |  |
| School health services | AND ("school health" OR "school nursing" OR "school nurse" OR "school nurses" OR "school mental") |
|  |  |
| Mental illness | AND (mental* OR mental OR mentally OR psych* OR psychological OR psychology OR psychiatric OR psychiatry) |
|  |  |
| Information and communication technology | AND (ICT OR "information technology" OR "communication technology" OR "communications technology" OR digital* OR digital OR digitalization OR digitalisation OR digiti* OR digitization OR digitisation OR comput* OR computer OR computers OR online* OR online OR internet* OR internet OR web OR WWW OR smartphone* OR smartphone OR smartphones OR "smart phone" OR "smart phones" OR iphone* OR iphone OR iphones OR mobile* OR mobile OR mobiles OR cellphone OR cellphones OR cellphone* OR "cell phone" OR "cell phones" OR "cellular phone" OR "cellular phones" OR tablet* OR tablet OR tablets OR ipad* OR ipad OR ipads OR software* OR software OR softwares OR app OR apps OR application OR applications OR eHealth OR "e-health" OR "e health" OR "electronic health" OR informatics) |
